# MARIDA: A benchmark for Marine Debris detection from Sentinel-2 remote sensing data

**DOI:** 10.1371/journal.pone.0262247

**Published:** 2022-01-07

**Authors:** Katerina Kikaki, Ioannis Kakogeorgiou, Paraskevi Mikeli, Dionysios E. Raitsos, Konstantinos Karantzalos

**Affiliations:** 1 Remote Sensing Laboratory, National Technical University of Athens, Athens, Zografou, Greece; 2 Institute of Oceanography, Hellenic Centre for Marine Research, Athens, Anavyssos, Greece; 3 Department of Biology, National and Kapodistrian University of Athens, Athens, Zografou, Greece; 4 Athena Research Center, Athens, Greece; Duy Tan University, VIET NAM

## Abstract

Currently, a significant amount of research is focused on detecting Marine Debris and assessing its spectral behaviour via remote sensing, ultimately aiming at new operational monitoring solutions. Here, we introduce a Marine Debris Archive (MARIDA), as a benchmark dataset for developing and evaluating Machine Learning (ML) algorithms capable of detecting Marine Debris. MARIDA is the first dataset based on the multispectral Sentinel-2 (S2) satellite data, which distinguishes Marine Debris from various marine features that co-exist, including *Sargassum macroalgae*, *Ships*, *Natural Organic Material*, *Waves*, *Wakes*, *Foam*, dissimilar water types (i.e., *Clear*, *Turbid Water*, *Sediment-Laden Water*, *Shallow Water*), and *Clouds*. We provide annotations (georeferenced polygons/ pixels) from verified plastic debris events in several geographical regions globally, during different seasons, years and sea state conditions. A detailed spectral and statistical analysis of the MARIDA dataset is presented along with well-established ML baselines for weakly supervised semantic segmentation and multi-label classification tasks. MARIDA is an open-access dataset which enables the research community to explore the spectral behaviour of certain floating materials, sea state features and water types, to develop and evaluate Marine Debris detection solutions based on artificial intelligence and deep learning architectures, as well as satellite pre-processing pipelines.

## Introduction

Marine Debris, such as plastics, is a major global issue with important environmental, economic, human health and aesthetic aspects. Plastics remain in the ocean for a long time, and have been found in various areas worldwide [1–3], affecting marine life at different trophic levels [4]. To tackle the Marine Debris issue, several solutions for detecting [5, 6], cleaning [7] and preventing [8] have been developed and validated. Among those, detecting and monitoring floating litter has recently gained the attention of most research and development efforts [9].

In particular, earth observation data from public and commercial satellite programs [10–14] have been employed for detecting and monitoring Marine Debris, as well as remote sensing data from manned aircraft [15], unmanned aerial vehicles (UAVs) [16–20], bridge-mounted [21] and underwater-cameras [22]. Spectral indices have also been proposed to enhance the detection of Marine Debris on multispectral satellite data, like the Floating Debris Index (FDI) [13] and the Plastic Index (PI) [23] that have been developed based on artificial plastic targets.

Furthermore, to better understand the spectral behaviour of Marine Debris, hyperspectral measurements have been conducted, exploring sensors’ capabilities in distinguishing plastics from other features such as vegetation, natural material, and water types [24–28]. Investigating Marine Debris characteristics (including its spectral behavior) has been also attempted via multispectral satellite observations [10, 12, 13, 29], highlighting that spectral discrimination of Marine Debris from other sea surface features (e.g., ships, foam) is not straightforward. Indeed, differentiating floating plastic debris from bright features, such as waves, sunglint, clouds, is currently considered very challenging [5, 6]. This is due to the fact that plastics have complex properties, diversifying in color, chemical composition, size and level of water submersion [30, 31]. A high-quality dataset can address the challenges mentioned above, supporting also the development and improvement of Marine Debris detection methods, and assessing the operational aspects of any given solution (e.g., scalability).

However, despite the challenging and continuously growing issue of Marine Debris, the currently available datasets are relatively limited in number and do not usually employ open-access high-resolution satellite data over geographically extended areas. These facts prohibit satellite data exploitation from ML frameworks and operational solutions. In addition, most of the currently available marine remote sensing datasets focus on detecting specific objects such as vessels [32–35]. Datasets for cloud detection over the ocean [36] and *Sargassum* macroalgae extraction [37, 38] have also been developed with a limited number of classes.

To this end, this study aims to fill this gap with a new, open-access benchmark dataset, named MARIDA—MARIne Debris Archive, based on S2 multispectral satellite data. MARIDA offers real cases with Marine Debris events, providing globally distributed annotations, ready for ML tasks. The produced dataset takes an innovative step forward by containing sea features that co-exist in remote sensing images, ultimately forming 15 thematic classes in total. Along with MARIDA, ML baselines for the weakly supervised semantic segmentation task [39] are presented, including shallow ML and deep neural network architectures. To enlarge the benchmark application area, the multi-label classification task is also considered.

## Materials and methods

### Dataset specifications

MARIDA is an open-source dataset consisting of annotated georeferenced polygons/pixels on S2 satellite imagery. MARIDA was designed to be temporally and geographically well-distributed; thus, we used open-access data from the S2 satellite sensor which coverage includes global coastal waters. S2 is capable of detecting and continuous monitoring large floating debris, as it provides multispectral data at a spatial resolution of 10 m and 20 m with a frequent revisit time of 2–5 days.

Regarding Marine Debris ground-truth data, reported events were collected from citizen scientists and social media over coastal areas and river mouths. After identifying these cases in S2 satellite data, the events were verified with very high-resolution satellite data (whenever possible due to availability), and the corresponding Marine Debris pixels were annotated. Additionally, sea surface features that co-occurred on satellite images were annotated: *Ships*, *Sargassum macroalgae*, *Foam*, *Waves* and *Natural Organic Material* (i.e., vegetation and woody), water types (i.e., *Clear*, *Turbid Water* and *Sediment-Laden Water*), *Shallow Coastal Waters* including benthic habitats, *Clouds* and *Cloud Shadows*. Regarding the annotation procedure, three image-interpretation experts annotated the satellite images by assessing the spectral and spatial patterns of all features, considering the limitations of the S2 sensor (i.e., different band resolutions and limited signal-to-noise ratio) [40]. Finally, an inter-annotator agreement protocol was established to merge the annotated data and aggregate the confidence levels derived from the three experts (see the Annotation process and protocol section).

The current benchmark dataset aims to support real-world scientific issues that could eventually not only facilitate research efforts in Marine Debris, but also offer operational monitoring solutions. Thus, MARIDA consists of realistic, non-iconic and non-ideal (e.g., with term ideal, we refer to cloud-free data during calm sea state conditions) satellite observations. MARIDA’s annotations are also sparse to reduce the potentially noisy labels due to the complexity of sea surface features. The annotated polygons with real cases on S2 images (10 m resolution) do not correspond to thematic class endmembers or pure/clear pixels (in some cases, we annotated sparse Marine Debris pixels or floating materials pixels under very thin clouds).

### Data collection and annotation

**Fig 1:**
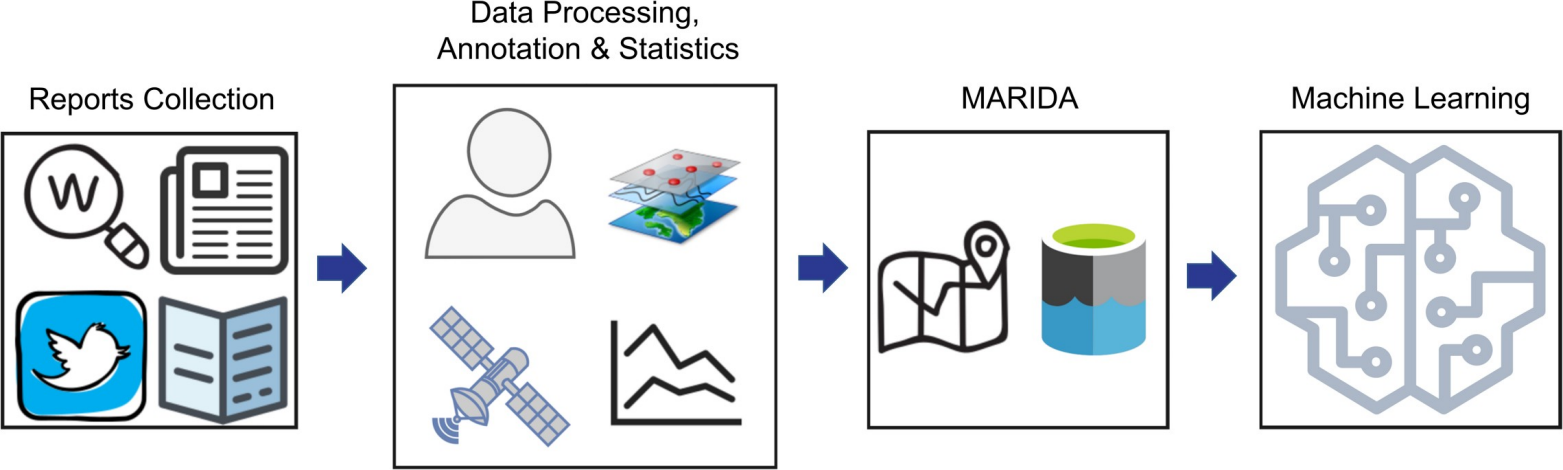
Schematic diagram representing the different steps for the construction of Marine Debris Archive-MARIDA.

For constructing MARIDA, a specific process was designed and followed, including three major steps (Fig 1): i) collection of reports (ground-truth data and literature) regarding floating Marine Debris events in coastal areas, ii) satellite data acquisition and processing, auxiliary weather data collection, spectral indices calculation, image interpretation and annotation, statistical analysis, and iii) MARIDA dataset generation and ML benchmarking.

#### Marine Debris reports

For a seven-year period (2015–2021), we gathered reports on marine litter and plastic pollution across coastal areas and river mouths in several countries (Table 1). The reports included observations gathered by photographers and citizen scientists, and information extracted from media, social media, and ocean clean-up activities. The URLs of the reports used are included in the S1 Table.

**Table 1:** Collected Marine Debris reports across different countries and continents for the period 2015–2021. The table shows the regions along with the reported events information (source, date and exact location).

Continent/ Country	S2 Tile	Source	Date	Location (WGS’84)
C. America/ Guatemala	16PCC	Citizen Scientist	18/9/20	15.836206° N, 88.022087° W
C. America/ Guatemala	16PCC	Photographer	16/6/18	15.827222° N, 88.047500° W
C. America/ Guatemala	16PCC	Kikaki et al. (2020)	4/9/19	14.9827° N, 89.5442° W
C. America/ Honduras	16PDC	Citizen Scientist	18/9/20	16.1490° N, 87.6282° W
C. America/ Honduras	16PEC, 16QED	Citizen Scientist	23/9/20	16.042194° N, 86.432081° W
C. America/ Honduras	16PEC	Kikaki et al. (2020)	29/11/15	16.0667° N, 86.3965° W
N. America/ S. Domingo	19QDA	Media	13/7/18	18.467723° N, 69.886808° W
N. America/ Haiti	18QWF/ QYF/ QYG	4ocean Clean-Ups	20/3/20	-
N. America/ Haiti	18QWF/ QYF/ QYG	4ocean Clean-Ups	5/1/21	-
N. America/ Haiti	18QWF/ QYF/ QYG	4ocean Clean-Ups	9/12/20	-
N. America/ Haiti	18QWF/ QYF/ QYG	4ocean Clean-Ups	15/12/20	-
Asia/ Indonesia	50LLR	Social Media	4/3/18	8.715828° S, 115.446799° E
Asia/ Vietnam	48PZC	Social Media	23/11/19	15.994762° N, 108.27417° E
Asia/ Philippines	51PTS	Social Media	18/5/19	-
Asia/ Philippines	51PTS	Social Media	16/7/16	-
Europe/ Scotland	30VWH	Biermann et al. (2020)	20/4/18	-
Africa/ South Africa	36JUN	Biermann et al. (2020)	24/4/19	-
Asia/ South Korea	52SDD	Jang et al. (2014)	-	-
Asia/ Indonesia	48MXU/ MYU	Cordova & Nurhati (2019)	-	-
Asia/ China	51RVQ	Zhao et al. (2019)	-	-

In addition to ground-truth data collection, the MARIDA dataset also included published satellite-derived data on Marine Debris detection [10, 13], and observations from rivers that have been reported in the literature as major polluters [2, 41–45]. Table 1 demonstrates the source of the reported data (i.e., ground-truth and indicated by literature), as well as the corresponding date and location, when available. For each area, corresponding S2 tiles are also included (Table 1).

#### Satellite data

Based on the ground-truth events, the corresponding S2 level1C images were acquired from Copernicus Hub (https://scihub.copernicus.eu/) for the exact reported dates and locations using a mean time window of 10 days. Additionally, for the regions that are significantly affected by plastic pollution (such as river discharges), the seasonality and the periods of maximum plastic presence were examined. We also extended our research for the entire 2015 to 2021 period, focusing on the major recorded rainfalls (https://power.larc.nasa.gov/data-access-viewer/).

At an early stage for selecting images with potential Marine Debris, we visually inspected S2 Red-Green-Blue (RGB) composites along with very high-resolution Planet (https://www.planet.com) and Google Earth imagery, when available (see S2 and S3 Tables). The S2 data in which the visual inspection indicated Marine Debris occurrence were further processed. Rayleigh reflectance values were extracted at 10 m resolution for 11 bands using ACOLITE atmospheric processor [46], excluding Vapour (Band 9) and Cirrus (Band 10). To improve the accuracy of the following annotation step, FDI [13] and FAI [47] spectral indices were calculated.

#### Annotation process and protocol

During this step, three image-interpretation experts had access to the gathered data, including reports, S2, Planet satellite imagery, and computed spectral indices. The annotators digitized Marine Debris based on ground-truth events, considering S2 sensor limitations, and employing domain knowledge about its spectral behaviour [10, 12, 13, 29, 30, 40] and its accumulation patterns (i.e., fronts, marine litter windrows) [48]. A laborious and intensive image interpretation and manual assessment of each pixel were performed for all selected images leading to Marine Debris annotations at pixel level. In addition, diverse floating objects, sea state features, water types and clouds were annotated based on image interpretation and established spectral patterns [31, 49–53]. Wind data were also utilized (https://power.larc.nasa.gov/data-access-viewer/) to examine the possibility of whitecaps, which may appear similar to plastics in human eye [31].

Expert annotators recorded the thematic class and their confidence level for each digitized polygon. In particular, all annotated polygons were labelled with three confidence levels (i.e., #1 for high confidence, #2 for moderate and #3 for low confidence level). After the annotation step, an inter-annotators agreement protocol was established, which is described below:

- For Marine Debris, *Natural Organic Material* and *Sparse Sargassum*, which occasionally can have similar spectral behaviour [40], the intersection per two annotators extracted (i.e., an agreement between at least two annotators regarding the class label). If so, the lowest confidence level that was originally assigned was kept for these cases.
- For the other features, the union of the annotated data was calculated. If at least two contradictory annotated classes existed for the same digitized area, the annotation was excluded. For the rest of the cases, where the three experts agreed regarding polygon labeling, the lowest confidence score was kept.

For each annotation, Marine Debris report existence was also recorded (i.e., #1 when exact date and locations were identified and matched to the available reports, #2 when patches were identified at a distance of either up to 20km or up to 6 days apart from the reported locations and dates; and #3 for no recorded reports close to the detected debris). Additionally, the cases that debris was detected based on previous studies reporting river discharges, were labelled under category 3 (Table 1).

For further details regarding our annotation strategy (cloud annotation and cases with floating materials and thin clouds interference) the reader is referred to the S1 Appendix.

#### Refining data

In order to improve the quality of our annotated data, the structure of the recorded high-dimensional observations (i.e., 11 multispectral bands) was visualized and explored. Specifically, to examine the pairwise distances between the high-dimensional annotated pixels, we utilized t-distributed Stochastic Neighborhood Embedding (t-SNE) algorithm proposed by Van der Maaten [54], using Spectral Angle Mapping (SAM) [30, 40, 55] as a distance metric. By representing our data in a 2D space, spectral patterns of thematic classes were mapped and outliers were identified and further explored (revisit the data to determine if they had been erroneously annotated).

The annotation procedure resulted in a vector dataset of the digitized polygons, in shapefile format. The dataset was converted into a raster structure, which was finally cropped into non-overlapping 256x256 pixel-sized patches. After the cropping, each patch was available for extra visual inspection.

### Machine learning frameworks

#### Baselines

In order to trigger more research efforts towards Marine Debris detection methods and solutions, we provide software baselines for *weakly supervised* pixel-level semantic segmentation tasks, by employing a Random Forest model (RF) [56] and an U-Net architecture [57].

In particular, RF is a well-established supervised model, which has been widely used in remote sensing and computer vision community. A RF classifier consists of many decision trees and uses averaging to improve the predictive performance and control over-fitting. For our RF model, we extracted features similar to the first place team of Track 2 of the 2020 IEEE GRSS Data Fusion Contest [58]. We trained three different RF models: i) one based on spectral signatures of each pixel (RF_SS_), ii) one based on spectral signatures and calculated spectral indices (RF_SS+SI_), and iii) one with spectral signatures, spectral indices, and extracted Gray-Level Co-occurrence Matrix (GLCM) [59] textural features (RF_SS+SI+GLCM_) in order to incorporate the spatial information. The extracted spectral indices were NDVI, NDWI, FAI, FDI, Shadow Index (SI), Normalized Difference Moisture Index (NDMI), Bare Soil Index (BSI) and NRD [40, 60], which are broadly used in remote sensing studies. To compute the GLCM features, Rayleigh corrected RGB composites were converted to grayscale images which consequently were quantized in 16 bins-level. The selected GLCM features were Contrast (CON), Dissimilarity (DIS), Homogeneity (HOMO), Energy (ENER), Correlation (COR) and Angular Second Moment [59]. For those features extraction, a window of size 13 x 13 was used.

The U-Net is a well-established deep learning model for semantic segmentation. Its architecture consists of two parts, the down-sampling and the up-sampling part. The first part encodes the input image yielding a low dimensional representation using successive blocks of 3 x 3 convolutions for features extraction and max-pooling layers for down-sampling. The feature maps/ produced channels are doubled in each block, while the spatial dimensions are reduced by half. The second part decodes the internal representation using successive up-convolution layers to create the final segmentation output.

For our task, the first input layer of U-Net was modified to adapt to the 11 Rayleigh reflectance S2 bands, and the final classification layer was changed to output the MARIDA classes. We also used 4 down-sampling and up-sampling blocks, as well as 16 hidden channels produced by the initial down-sampling block.

To assess pixel-level semantic segmentation performance, we relied on three metrics. Our main evaluation metric was the Jaccard Index or Intersection-over-Union (IoU) [61]. In addition, the average for each class *F*_1_ score (Macro-*F*_1_/ *mF*_1_) and the Pixel Accuracy (PA) for the per-class assessment were employed (S2 Appendix).

Through MARIDA, we also provide multi-labels in patch-level, which formulate a weakly-supervised multi-label classification task with positive, and absent labels that are not necessarily negative [62, 63]. For the baseline of the multi-label classification task, we adopted the Residual neural network (ResNet) [64]. The evaluation metrics for the multi-label classification task are demonstrated in the S2 Appendix and the proposed baseline in the S4 Appendix.

### MARIDA dataset and analysis

**Fig 2:**
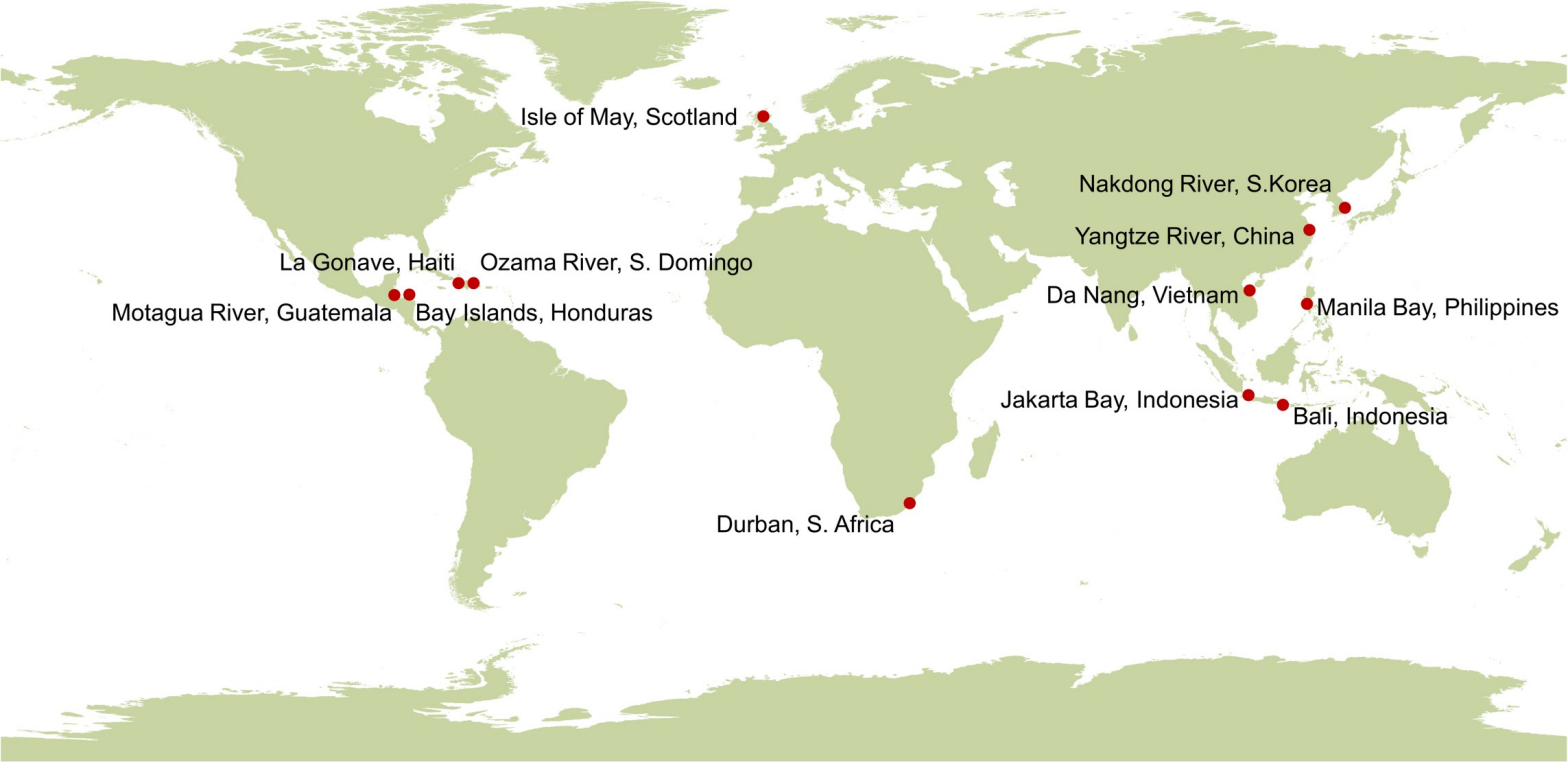
The sites (red dots in the map) where Marine Debris events were reported, and corresponding Sentinel-2 satellite images were acquired and processed. Marine Debris and other features that co-existed were annotated in considered satellite data. The corresponding map is acquired from Natural Earth (http://www.naturalearthdata.com/).

MARIDA contains 1381 patches, consisting of 837,357 annotated pixels, based on 63 S2 scenes acquired from 2015 to 2021. MARIDA provides patches with corresponding masks of pixel-wise annotated classes and confidence levels in the format of GeoTiff. For each patch, the assigned multi-labels are given in a JSON file. In addition, MARIDA includes shapefiles data in WGS’84/ UTM projection, with file naming convention following the below scheme: s2_dd-mm-yy_ttt, where s2 denotes the S2 sensor, dd denotes the day, mm the month, yy the year and ttt denotes the S2 tile. Shapefiles data include the class of each annotation, along with the confidence score and the report description. The produced dataset is composed of geodata, covering different sites around the globe (Fig 2). The selected study sites are distributed over eleven countries (i.e., Honduras, Guatemala, Haiti, Santo Domingo, Vietnam, South Africa, Scotland, Indonesia, Philippines, South Korea and China).

### Thematic class distribution

**Fig 3:**
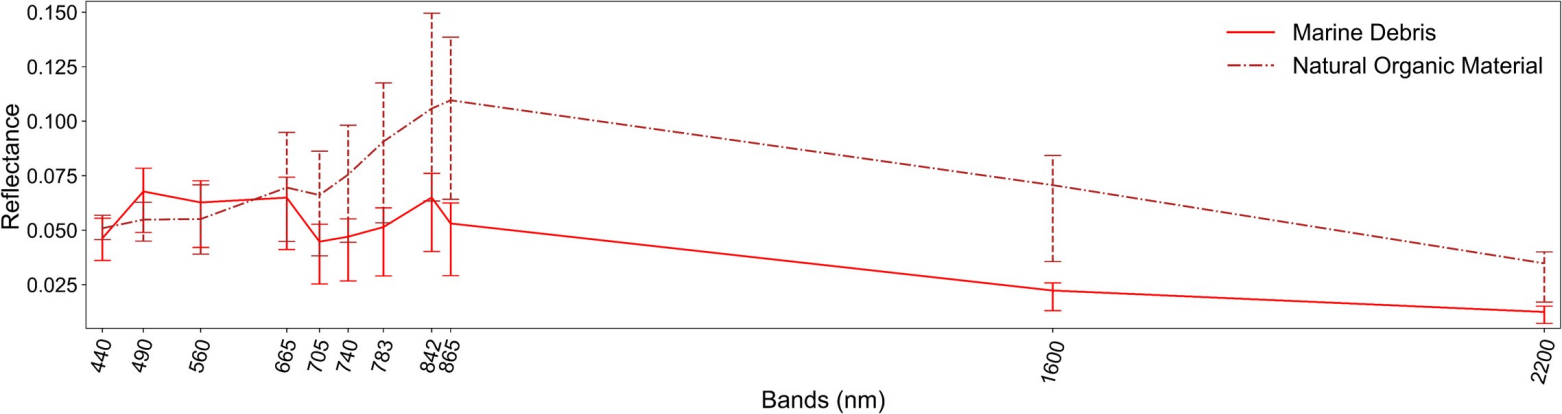
The spectral signatures of the Marine Debris and *Natural Organic Material* classes derived from the annotations with the high confidence levels. The mean spectral signatures are presented with 25–75 percentiles as error bars.

**Fig 4:**
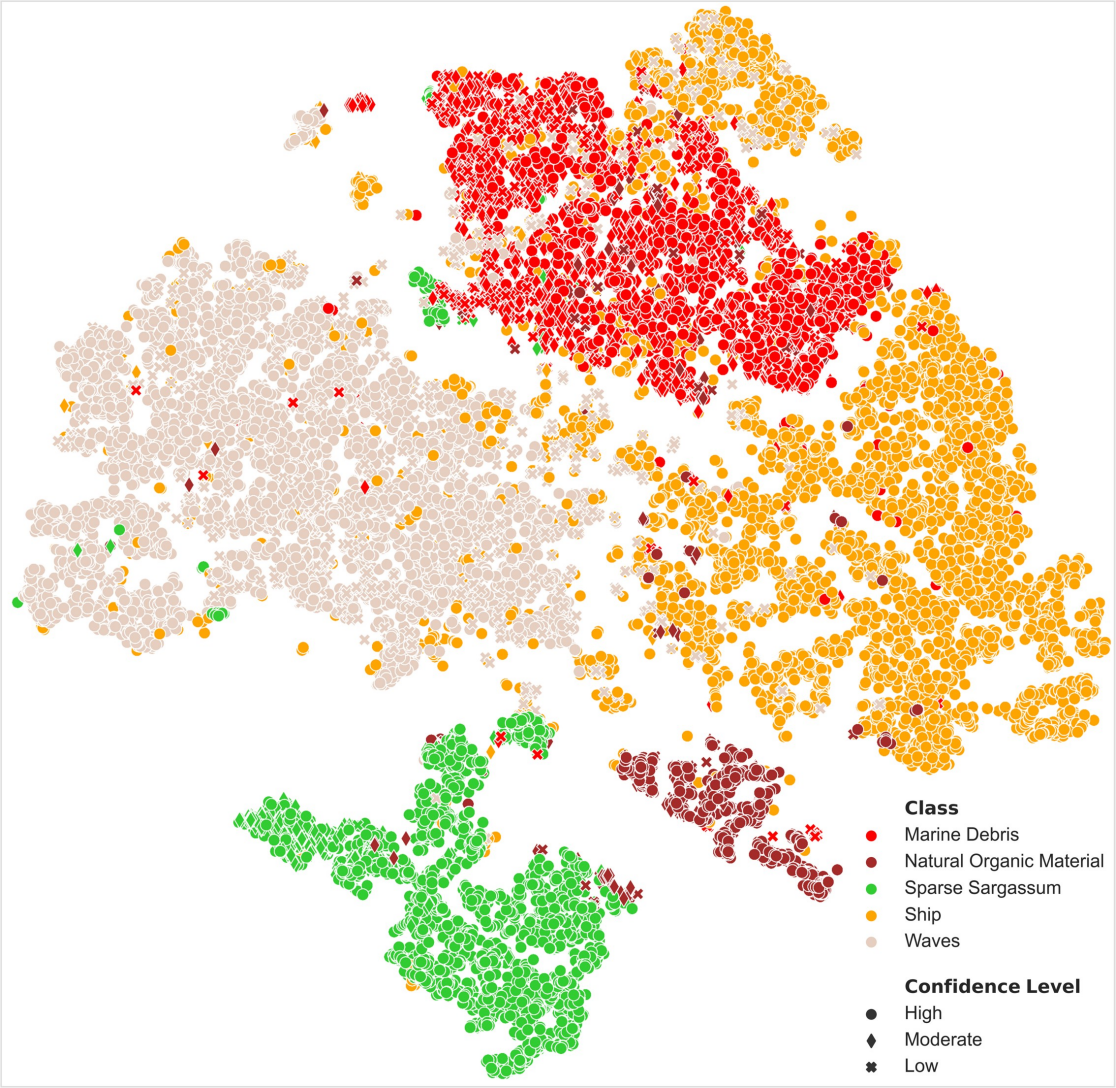
A 2D embedding using T-SNE algorithm with SAM metric for the classes: Marine Debris, Ships, Sparse *Sargassum*, *Natural Organic Material* and Waves. Each class is represented with a different color. Different symbols demonstrate the confidence level of annotations.

To demonstrate the descriptive overview of MARIDA, the class and pixel distributions are presented in Tables 2 and 3 and their spectral and statistical analysis are illustrated in Figs 3 and 4. More specifically, the 15 different classes of MARIDA are shown in Table 2, which includes the class description, the corresponding number of provided image patches, and all acronyms of the annotated classes. Regarding the class distribution, the *MWater* class has been digitized in 870 patches due to its implicit abundance in satellite data and straightforward annotation. As proposed by Hu [40], we have included additional *MWater* pixels that were close to Marine Debris pixels, in order not only to facilitate further experiments with SAM, but also run experiments on pixel windows (3x3 or 5x5) and reflectance difference. The second-highest number of 373 patches were labelled as Marine Debris, indicating the high variety of annotations in different patches. *Cloud*, *Ship* and *Turbid Water* were annotated in a sufficient number of patches (~200), as they are plenty in the natural environment and easily identified by annotators.

**Table 2:** The thematic classes of MARIDA. Name, description and corresponding number of patches are presented for each class. All acronyms are stated here.

Class Name	Acronym	Description	Number of Patches
Marine Debris	MD	Floating plastics or other polymers, mixed anthropogenic debris	373
Dense *Sargassum*	DenS	Dense floating *Sargassum* macroalgae	49
Sparse *Sargassum*	SpS	Sparse floating *Sargassum* macroalgae	106
Natural Organic Material	NatM	Vegetation & Wood	71
Ship	Ship	Sailing & Anchored Vessels	182
Clouds	Cloud	Clouds including thin Clouds	181
Marine Water	MWater	Clear Water	870
Sediment-Laden Water	SLWater	High-Sediment river discharges with brown colour	51
Foam	Foam	Foam recorded at river fronts or coastal wave breaking area	59
Turbid Water	TWater	Turbid waters close to coastal areas	220
Shallow Water	SWater	Coastal waters, including coral reefs and submerged vegetation	64
Waves	Waves	Waves	54
Cloud Shadows	CloudS	Cloud Shadows	71
Wakes	Wakes	Wakes & Waves from a sailing vessel	106
Mixed Water	MixWater	Water near floating materials	140
**Total**			**1381**

**Table 3:** MARIDA’s class distribution at pixel-level. For Sentinel-2 tiles description, the reader is referred to Table 1. For classes acronyms, the reader is referred to Table 2.

S2 Tile	MD	DenS	SpS	NatM	Ship	Cloud	MWater	SLWater	Foam	TWater	SWater	Waves	CloudS	Wakes	MixWater	# of pixels	# of S2 scenes
16PCC	1496	2048	574	78	3322	62082	60169	285886	712	99501	3960	3417	3585	5929	191	532950	19
16PDC	143	49	226	78	96	13507	15258	85449	334	24923	2251	0	883	253	75	143525	6
16PEC	129	222	645	193	485	11678	19341	11	86	27080	3782	108	1733	1115	51	66659	6
16QED	0	474	691	0	90	4098	1719	0	0	0	5910	0	1841	221	0	15044	2
18QWF	0	0	0	0	0	0	324	0	0	0	0	1461	0	0	0	1785	1
18QYF	1112	4	200	154	408	7977	1360	0	0	0	1038	0	314	48	58	12673	13
18QYG	90	0	0	7	0	373	222	0	0	831	277	0	106	0	15	1921	1
19QDA	0	0	21	3	11	0	110	0	0	5	40	0	0	0	0	190	1
30VWH	27	0	0	0	36	3505	24393	0	0	0	0	0	1975	0	0	29936	1
36JUN	46	0	0	0	625	3500	600	0	0	300	0	0	0	18	0	5089	1
48MXU	208	0	0	0	71	5807	194	0	0	382	45	0	489	15	12	7223	2
48MYU	24	0	0	0	223	0	291	0	0	10	48	0	0	611	0	1207	2
48PZC	24	0	0	0	298	4108	2079	0	48	4129	0	0	765	171	1	11623	3
50LLR	41	0	0	3	27	402	485	0	41	0	18	841	0	72	5	1935	1
51PTS	38	0	0	20	17	0	35	0	0	0	0	0	0	0	0	110	2
51RVQ	17	0	0	0	0	363	163	0	0	0	0	0	37	0	0	580	1
52SDD	4	0	0	328	94	0	2416	1591	4	451	0	0	0	37	2	4927	1
**Total pixels**	3399	2797	2357	864	5803	117400	129159	372937	1225	157612	17369	5827	11728	8490	410	**837377**	**63**
**Perc. %**	0,41	0,33	0,28	0,1	0,69	14,02	15,42	44,54	0,15	18,82	2,07	0,70	1,40	1,01	0,05	100	

The rest of the categories were digitized in fewer patches (appr. 50–100). Some of the considered categories, such as *SLWater*, *Sargassum* blooms, *CloudS*, *SWater* were easily digitized with compact, not extended polygons, while *Foam*, *NatM*, *Wakes* and *Waves* required a laborious and intensive manual assessment. Considering that MARIDA is a Marine Debris-oriented dataset, we provide only a certain number of indicative cases with the classes mentioned above. The artifact due to the dissimilar S2 band resolutions led to a specific spectral signature primarily recorded on surrounding water pixels of Marine Debris, *SpS* and *Ship*. This class was labelled as *MixWater*, as it corresponds to water, and digitized around annotated Marine Debris pixels. For more details about patches and class co-occurrence, readers are referred to the online material (https://marine-debris.github.io/).

Apart from the per-patch analysis, we also discuss the pixel-level distribution of MARIDA classes. Table 3 summarizes the class distribution in pixel level for each S2 tile, indicating that MARIDA provides numerous pixels annotated in 17 S2 tiles. Overall, most given pixels correspond to Honduras Gulf, a known plastic polluted region where a thorough remote-sensing study has been previously conducted by Kikaki et al. [10], based on ground-truth data. It should be noted that, although we avoided digitizing extended regions with water or clouds, the produced dataset cannot be balanced at pixel-level due to the implicit different size and characteristics of considered sea features. Indeed, our goal was to create a Marine Debris-oriented dataset.

To this end, we provide a significant number of 3339 Marine Debris pixels in total. The 1625 pixels were digitized and annotated with high confidence, based on reports and domain knowledge. Additionally, 1235 pixels were labelled with moderate and 539 pixels with low confidence (S4 Table). For scenes with large garbage trajectories and high confidence annotations, the readers are referred to 18 September 2020 (tile 16PCC) and 14 March 2020 (tile 18QYF), where ground-truth events were available. An indicative case with dense marine litter patches at Motagua river mouth was also evident on 4 September 2016 (tile 16PCC). For other scenes with high-confidence Marine Debris annotated data, the reader can consider the online material (https://marine-debris.github.io/).

### Spectral signatures

To study the spectral behavior of Marine Debris annotated data, we extracted the mean spectral signatures for each scene, leading to a detailed analysis presented thoroughly in the online material. The mean spectral reflectance of annotated pixels with high confidence in MARIDA is depicted in Fig 3. The mean spectral signatures are presented along with 25–75 percentiles as error bars to demonstrate the variation along with the skewness of their distribution. Atmospheric correction process, diverse proportions of floating Marine Debris within pixels, differences resulting from colours and immersion, and mixed conditions in the natural environment led to high variability of recorded Marine Debris spectral signatures.

However, the recorded Marine Debris mean spectral reflectance is very similar with the corresponding simulated signature proposed recently by Hu [40]. Slightly higher values in our data indicate different debris proportions within pixels. In comparison with previous studies [10, 12, 13], which exploited S2 imagery, higher reflectance at Green and Red bands was observed, possibly due to the denser patches that we recorded. Additionally, the mean spectral signature of high-confidence *NatM* was considered for comparison, as in some cases with low subpixel proportions, their spectral discrimination was not straightforward. Regarding Marine Debris and *NatM* comparison, it was found that their discrimination might be possible in 865 nm and SWIR bands.

### Statistical analysis

By applying t-SNE algorithm along with spectral signatures analysis described above (Figs 3 and 4, online material), important insights were gained about spectral behaviour of floating Marine Debris and the potential of spectral discrimination from other features with similar patterns such as *SpS*, *Ship*, *Waves* and *NatM*.

Fig 4 presents t-SNE results for the considered features, indicating the different confidence level for each annotation with a different symbol. Based on the recorded data, a well-shaped Marine Debris cluster was developed, which is discrete from other clusters. Very sparse recorded Marine Debris (e.g., 20 April 2018 in Scotland) led to a smaller separate cluster between *Waves* and Marine Debris. A well-shaped *Ship* cluster was also mapped, yet some annotated *Ship* pixels were depicted in Marine Debris cluster due to the similar polymer types. Respectively, some dense Marine Debris pixels were mapped in the *Ship* cluster. Some *Ship* pixels were also depicted close to *Waves* pixels; this is evident in cases with moving vessels, where discrimination of boundary *Ship* pixels from water-related classes (i.e., *Wakes*) was challenging for a human expert.

Occasionally, *NatM* cannot be spectrally separated from Marine Debris (e.g., 18 September 2020 at Motagura river mouth). Mixed conditions at the river mouth, low coverage at pixel-level and potentially colored marine litter (e.g., green or brown) led to uncertainties represented with low confidence Marine Debris and *NatM* annotations. However, dense *Natural* woody debris has a discrete spectral signature (e.g., 7 October 2018 at Nakdong river mouth). This fact was also confirmed by a smaller (but well-shaped) *NatM* cluster depicted in brown color (Fig 4). A discrete *SpS* cluster was also formed, including *NatM* (i.e., vegetation). In some cases the *SpS* annotated pixels have been mapped in the Marine Debris and *Waves* clusters, though, the majority of these cases corresponded to sparse floating materials that were detected at a lower subpixel level. This fact confirms that sparse floating vegetation pixels in some cases cannot be spectrally discriminated from sparse marine litter pixels (e.g., 4 March 2018 in Bali) [40].

### MARIDA benchmark and ML baselines

MARIDA is designed to be beneficial for several remote sensing applications and tasks which are described in detail in the following section (Discussion). However, it primarily aims to benchmark weakly supervised pixel-level semantic segmentation learning methods. In particular, the produced dataset falls into incomplete-supervision due to sparsely annotated data, inexact-supervision due to sensor limitations (i.e., 10 m resolution, different bands resolution), and inaccurate supervision derived from potential slightly noisy annotations (i.e., sensor noise, human error) [40].

### Dataset split and training procedure

MARIDA was split into train, validation and test disjoint sets. The data were not split randomly; instead, each data split was produced as a representative subset of the whole dataset. For instance, the dataset was divided into subsets which were ensured to have balanced class distribution (S5 Table). It should be noted that the data of each scene/unique date were retained in the same set. The split was selected to be ~50/25/25%. More specifically, the split contains 694 training (429,412 px), 328 validation (213,102 px) and 359 test (194,843 px) patches.

Due to the moderate size of MARIDA and aiming at a Marine Debris-oriented dataset, the initial 15 classes were aggregated to 11 classes. The categories of *Wakes*, *CloudS*, *Waves* and *MixWater* were grouped with *MWater* and formed a water super-class, as they semantically belong to the same class as well as present similar spectral profiles (see online material).

Regarding RF training, all models (RF_SS_, RF_SS+SI_, RF_SS+SI+GLCM_) were composed of 125 trees, each with a maximum depth of 20 nodes. Due to pixel-level class distribution, which is by nature imbalanced (e.g., *Marine Water* px contrary to Marine Debris px), we used class weighting inversely proportional to class frequencies in the training set. Additionally, the annotators’ confidence score was utilized such that low confidence samples contribute less to the training process. Specifically, the weights for high, moderate and low confidence samples were 1, 2/3 and 1/3, respectively. The final selection of RF hyperparameters described above was based on grid search in the validation set.

During the U-Net training process, the Adam algorithm was employed to minimize the Cross-Entropy loss with an initial learning rate of 2x10^-4^. Moreover, we utilized early stopping based on the loss of the validation set and trained for 44 epochs. After the 40^th^ epoch, the learning rate was reduced to 2x10^-5^. The selected batch size was 5 samples. We also employed random rotations of the input images by -90°, 0°, 90°, or 180° and horizontal flips in order to augment the dataset. The selection of the hyperparameters above and training set-up was based on grid search in the validation set. It should be noted that the U-Net model was trained from scratch. A weighting scheme on the Cross-Entropy loss was also utilized, to address the unbalanced data issue [65] (S3 Appendix). Finally, it should be mentioned that in our U-Net baseline, in contrast to RF, we did not experiment with the annotators’ confidence levels.

### Baseline experiments and evaluation

This subsection describes the quantitative and qualitative assessment of our ML baseline outcomes in MARIDA. To evaluate our results quantitatively, we demonstrate the scores for all metrics per class on the test set (Table 4). Overall, our results indicate that RF_SS+SI+GLCM_ leads to the highest average scores for all metrics, followed by RF_SS+SI_ and RF_SS_, which provide almost equivalent average scores. Regarding scores per class, for *SWater*, U-Net provides the highest scores, while for *Ship*, *Clouds*, *MWater* and *Foam*, RF_SS+SI+GLCM_ performs best. For *DenS*, RF_SS+SI_ leads to the highest scores, as for *SpS*, RF_SS+SI+GLCM_ leads to higher scores for IoU and F_1_. For *TWater*, both RF_SS+SI+GLCM_ and U-Net achieve similarly high scores. It is noteworthy to highlight that for *SLWater*, all RF models and U-Net achieve for all metrics the highest scores (i.e., 1).

**Table 4:** Evaluation scores obtained by RF_SS_, RF_SS+SI_, RF_SS+SI+GLCM_ and U-Net for each class on Marine Debris Archive. The highest scores are highlighted. All acronyms are stated in Table 2.

	RF_SS_			RF_SS+SI_			RF_SS+SI+GLCM_			U-Net		
Class	IoU	PA	F_1_	IoU	PA	F_1_	IoU	PA	F_1_	IoU	PA	F_1_
MD	0.55	0.91	0.71	**0.67**	**0.92**	**0.8**	0.65	**0.92**	0.79	0.33	0.7	0.5
DenS	0.87	0.92	**0.93**	**0.88**	**0.93**	**0.93**	0.87	**0.93**	**0.93**	0.6	0.6	0.75
SpS	0.53	0.91	0.69	0.69	**0.92**	0.82	**0.83**	0.9	**0.91**	0.66	0.89	0.79
NatM	**0.31**	**0.47**	**0.47**	0.17	0.27	0.29	0.18	0.31	0.31	0.02	0.02	0.04
Ship	0.54	0.72	0.7	0.47	0.7	0.64	**0.67**	**0.82**	**0.8**	0.62	0.76	0.76
Clouds	0.75	0.85	0.86	0.74	0.82	0.85	**0.84**	**0.86**	**0.91**	0.62	0.62	0.76
MWater	0.66	0.82	0.79	0.65	0.83	0.79	**0.75**	**0.93**	**0.86**	0.61	0.88	0.76
SLWater	**1**	**1**	**1**	0.99	**1**	**1**	0.99	**1**	**1**	0.99	0.99	**1**
Foam	0.23	0.29	0.37	0.31	0.48	0.47	**0.6**	**0.74**	**0.75**	0.55	0.55	0.71
TWater	0.74	0.78	0.85	0.8	0.83	0.89	**0.88**	0.92	**0.94**	0.84	**0.95**	0.91
SWater	0.08	0.25	0.16	0.13	0.33	0.23	0.3	0.37	0.46	**0.45**	**0.67**	**0.62**
Average	0.57	0.72	0.69	0.59	0.73	0.7	**0.69**	**0.79**	**0.79**	0.57	0.69	0.69

Regarding Marine Debris, RF_SS+SI_ performs the highest scores, while adding spatial information does not improve the classification performance results (i.e., IoU and F_1_ decreased slightly). Future experiments with different window sizes for the extraction of GLCM textural features may lead to higher scores. We have to note that, for the *NatM* class, all models lead to low scores. *NatM* presents similar spectral behavior to Marine Debris, while both follow the same spatial patterns (e.g., linear trajectories). In this case, adding spectral indices or textural information leads to lower scores than the initial. Especially, U-Net predicts only few annotated *NatM* pixels on the test set.

**Fig 5:**
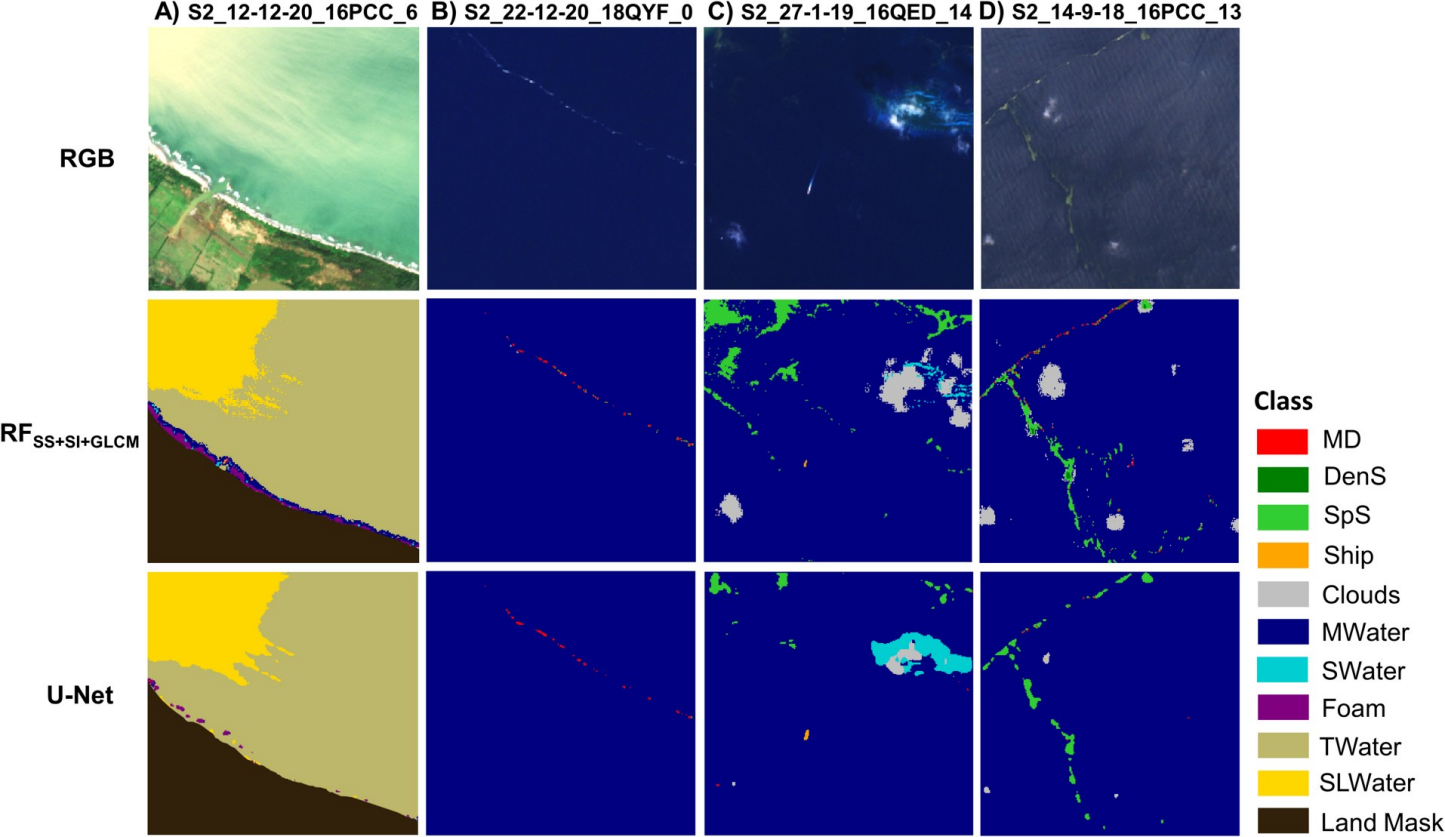
Classification results extracted by the baseline RF_SS+SI+GLCM_ and U-Net models. Selected indicative cases demonstrate (A) S2_12-12-20_16PCC_6, (B) S2_22-12-20_18QYF_0, (C) S2_27-1-19_16QED_14 and (D) S2_14-9-18_16PCC_13 patches on test set. RGB patches are derived from Sentinel-2 data which were freely downloaded from https://earthexplorer.usgs.gov/. All acronyms are stated in Table 2.

Except for the quantitative evaluation described above, a qualitative (visual) assessment of our baseline results on the test set was also performed (Fig 5). As it is easily noticed, the two models, RF_SS+SI+GLCM_ and U-Net, provide similar results. Nevertheless, U-Net seems more robust to S2 noise and single pixels with sharp spectral differences than RF. U-Net is capable of modeling the shapes and spatial patterns of sea features, and appeared to be no sensitive in isolated pixels/ spikes, potentially due to the inherent multiple-scale information (successive convolutional layers). On the other hand, RF_SS+SI+GLCM_ is more prone to S2 noise and mixed bands resolutions artifact. In particular, in RF_SS+SI+GLCM_ results, some pixels around Marine Debris and *SpS* are classified as *Cloud* (Fig 5B and 5D).

In both models, small vessels are classified as Marine Debris (Fig 5C), which is expected due to similar polymer types that are composed and possibly similar floating material proportion within pixel. Regarding *Cloud*, RF_SS+SI+GLCM_ predicts more accurately the considered class than U-Net (Fig 5C and 5D), while U-Net predicts better the *SWater* habitats (Fig 5C). The latter fact can be also seen in the highest scores in all U-Net metrics (Table 4). In the coastal zone, both models lead to similar results. However, in U-Net classification images, some *Foam* pixels are predicted as Marine Debris, while in RF results, some *TWater* pixels are classified as *MWater* (Fig 5A).

By assessing our baseline experiments quantitatively and qualitatively, we observe that there is a consistency between metric scores and classification outputs in general. Yet, in some cases, the classification is still challenging. For instance, although both models achieve high scores (in comparison with other classes) for *SpS* (Table 4), in some cases with very sparse conditions, *SpS* pixels are classified as Marine Debris (Fig 5D).

For the evaluation scores regarding the multi-label classification task (ResNet) the reader is referred to the S6 Table.

## Discussion and challenges

In this work, a new dataset (MARIDA) is introduced towards triggering the research community at improving and developing new methods for detecting Marine Debris and discriminating from other sea surface features that co-exist. Based on the collected ground-truth, literature review and intensive image interpretation, MARIDA provides 3399 Marine Debris pixels, labelled in different S2 tiles across various countries, different seasons, years and sea state conditions. Thus, MARIDA is an important geodata source for evaluating existing detection methods and developing new techniques based on available S2 data.

**Fig 6:**
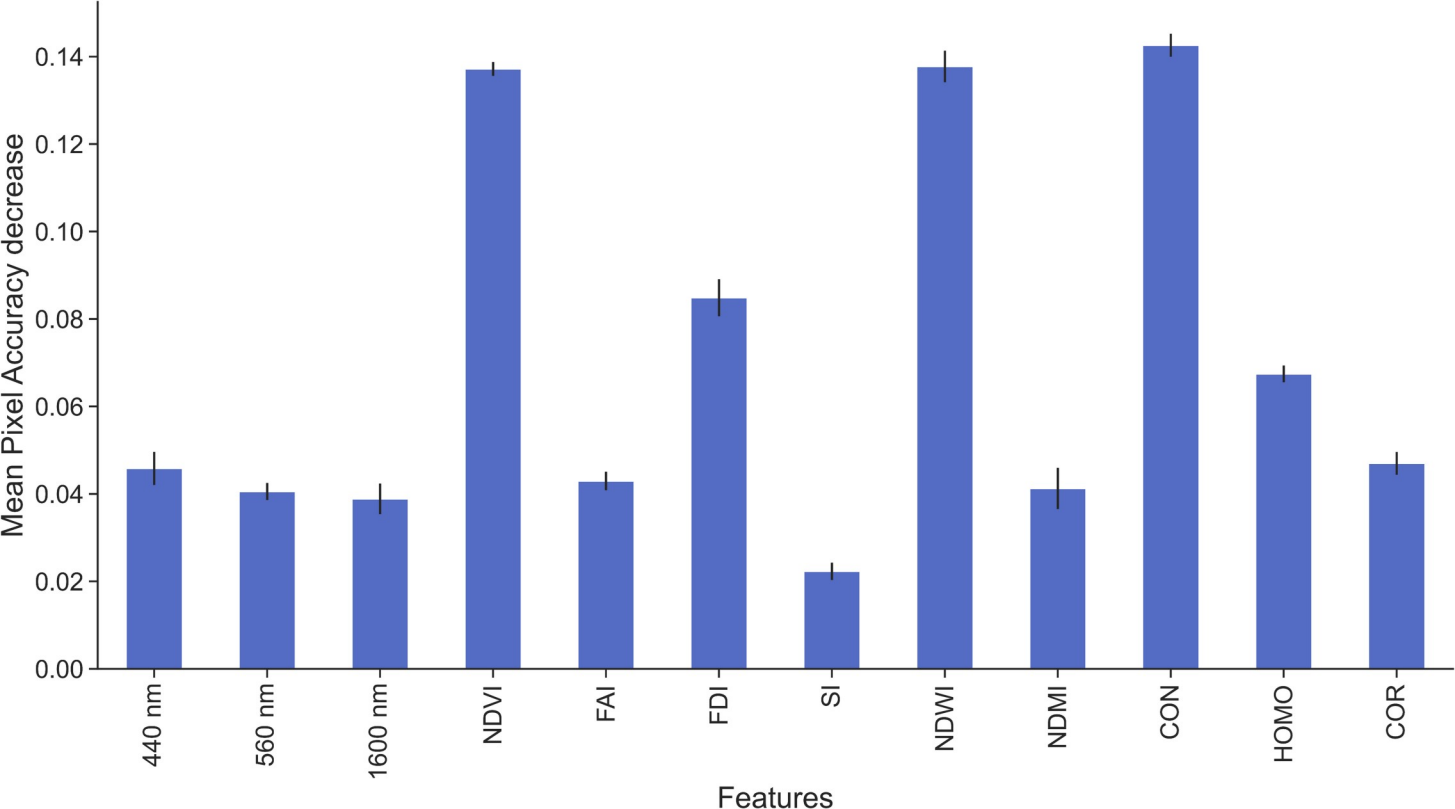
Features importance using permutation on RF_SS+SI+GLCM_ model. Each feature represents a different highly correlated group. The largest mean pixel accuracy decrease occurs by permuting CON, NDWI, NDVI and FDI.

After training four different models, the results showed that the developed RF_SS+SI+GLCM_ achieved the highest scores for all metrics; yet it seems more prone to S2 noise and different bands resolutions than the deep U-Net architecture. Further experimentation with RF_SS+SI+GLCM_ indicated that the most distinctive feature is the spatial feature CON (i.e, a measure of the intensity difference between a pixel and its neighbour), followed by NDWI, NDVI and FDI (Fig 6 and S5 Appendix and S1 Fig). This fact is also in line with Tasseron et al. [27] who recommended that the combination of FDI and NDVI can be efficient in the separation of vegetation and Marine Debris.

Low-confidence annotations were also included in our dataset, revealing challenging cases where no ground-truth events existed, and thus, human-experts attempted to identify the floating materials/ features based on domain knowledge, image interpretation and statistical analysis. Indicative cases include the sparse floating materials detected at fronts (e.g., 1 December 2019 in Jakarta Bay), very turbid conditions (e.g., 12 January 2017 in Honduras), and windrows (29 August 2017 at Yangtze river mouth) where human-experts could not easily define if they were dominated by dense foam or plastic concentrations. In addition, the spectral discrimination of Marine Debris from *NatM* was not straightforward in some cases (e.g., 18 September 2020 PCC). This issue was also observed in a previous study by Moshtaghi et al. [66], demonstrating that the considered floating materials (e.g., brown Marine Debris and woody debris) can have similar spectral patterns.

Regarding MARIDA limitations, it should be noted that the dataset is not optimally balanced geographically due to the lack of open-access in situ data reporting marine litter cases worldwide. MARIDA dataset can be augmented in future works with other datasets (e.g., clouds), other recorded features such as macroalgae species (e.g., *Ulva*, *Noctiluca*), jellyfish blooms [29] and future collections of additional verified Marine Debris events.

Due to S2 spatial resolution, the annotation procedure was occasionally not straightforward. For example, the discrimination between boundary *Ship* pixels and *Wakes* in moving ships was challenging for all experts. Thus, these cases potentially induced slight noise to the dataset. Certain S2 images with erroneous atmospheric corrections, such as the S2 image acquired on 23 September 2020 (Bay Islands, Honduras), were excluded, even though a major Marine Debris event was reported in the region during this date. Furthermore, high cloud coverage did not allow marine litter detection in all available S2 images in Santo Domingo, where a significant event was reported (July 2018).

The ACOLITE Dark Spectrum Fitting (DSF) algorithm was selected in this work after the recommendation from several studies [10, 12, 13, 40], reporting that ACOLITE performed well in detecting marine litter. However, ACOLITE performs simple pixel replication and no interpolation (such as bilinear or cubic) or other more sophisticated methods such as pan-sharpening to resample the S2 20 m and 60 m bands to 10 m.

Despite the limitations mentioned above, MARIDA is designed to be a multi-task dataset with various future aspects. Firstly, the RF model used here can be further enhanced by using spatial information at multiple scales (e.g., GLCM features at different windows size). Further feature-engineering and selection of the most distinctive bands, might improve the RF performance as well. Also, the experimentation with the denoising of the prediction masks (as a meta-classifier) can create more accurate classification outputs.

Regarding U-Net, experimentation with different loss functions and different weighting schemes can potentially address the class imbalance. For instance, the Focal Loss [67] may help the model focus on classes that have not been trained well. Furthermore, the exploitation of annotators’ confidence level information should be incorporated into the learning process. Another arising challenge is the combination of the predictions from multiple models (ensemble methods), potentially leading to more promising results. Experimentation with other improved or more sophisticated architectures can also be examined. The integration of advanced pre-processing techniques (i.e., cloud masking, denoising algorithms) should improve Marine Debris detection and sea features classification outcomes [37].

Beyond *weakly supervised* semantic segmentation, MARIDA can be re-used for several remote sensing and ML applications. One straightforward task, which is being proposed in the S4 Appendix, is the *weakly supervised* multi-label classification task (missing labels). Concerning this task, exploring different Curriculum Learning-based strategies for predicting missing labels [63] might be essential. In addition, experimentation with different loss functions can further improve the results. For instance, although the multi-class Cross Entropy loss (Softmax loss) is not tailored for multi-label settings and can be counter-intuitive, it often shows better results [68].

Other tasks derived by MARIDA that could be further explored are the unsupervised classification methods and/or clustering analysis, for better understanding the spectral patterns of sea features. In addition, the produced dataset can be used to evaluate existing spectral indices such as FDI, FAI and optimal thresholds tuning, as well as the development of new spectral indices. Last but not least, by providing annotated water pixels close to Marine Debris, we encourage the readers to further experiment with subtracting nearby water pixels (i.e., reflectance difference), windows-size and x subpixel proportion [40].

## Conclusions

In this work, we present MARIDA, a benchmark dataset for the detection of Marine Debris on S2 multispectral satellite data. MARIDA challenges the research community by: i) offering annotations of Marine Debris and various sea features that co-occur in realistic cases, ii) providing a detailed overview of MARIDA as well as spectral signatures analysis of annotated data, iii) evaluating ML algorithms, and iv) identifying application cases and open issues. Considering that marine litter research is increasing significantly and plastic debris monitoring using remote sensing is still challenging, we provide a Marine Debris dataset appropriate for future detection experiments and ML classification tasks. We envisage the continuous expansion of this dataset, including additional cases from the global oceans.

## Supporting information

S1 Table Source of Marine Debris reports with available links. All links were last accessed on 24 July 2021.
(PDF)

S2 Table The revisit time (days) of Sentinel-2 and Planet satellite sensors in study sites. (PDF)

S3 Table The acquisition dates (day/month/year) of Sentinel-2 satellite data used for MARIDA construction. Corresponding Planet data (photo-interpretation process) are presented.
(PDF)

S4 Table The distribution of the confidence scores in pixel level for the classes of Marine Debris, *Natural Organic Material* and *Sparse Sargassum*. (PDF)

S5 Table Class distribution (%) for each split in MARIDA. All acronyms are stated in Table 2.
(PDF)

S6 Table Evaluation scores obtained by ResNet for each class on multi-label classification. All acronyms are stated in Table 2.
(PDF)

S1 Appendix Cloud and floating material annotation. (PDF)

S2 Appendix The evaluation metrics. (PDF)

S3 Appendix Class weighting scheme. (PDF)

S4 Appendix ResNet baseline. (PDF)

S5 Appendix Features correlation and importance. (PDF)

S1 Fig Features correlation. (A) Agglomerative hierarchical clustering on Spearman Correlation. (B) Heatmap of features correlation.
(TIF)
