# Supplementary material for: MARIDA: A benchmark for Marine Debris detection from Sentinel-2 remote sensing data
Source: PLoS One. 2022 Jan 7;17(1):e0262247. doi: 10.1371/journal.pone.0262247 (PMC8740969; doi:10.1371/journal.pone.0262247)
Supplement: S1 Table — All links were last accessed on 24 July 2021. (PDF) [file pone.0262247.s001.pdf]

S1 Table. Source of Marine Debris reports with available links. All links were last accessed on 24 July 2021.

Site	Source	URL Link
Santo Domingo	Media	https://www.youtube.com/watch?v=sFy1Vmm42zQ
Indonesia/Bali	Social Media	https://www.youtube.com/watch?app=desktop&v=31CdhLMV7Es
Vietnam/ Danang	Social Media	https://www.tripadvisor.com/ShowUserReviews-g298085-d456220-r728403711-Non_Nuoc_Beach-Da_Nang.html
Haiti/ La Gonave Gulf	4ocean Clean-Ups	https://www.facebook.com/4oceanBracelets/
Philippines/ Manila Bay	Social Media	https://www.dreamstime.com/manila-philippines-may-ocean-plastic-pollution-manila-bay-shore-manila-philippines-may-ocean-plastic-pollution-manila-bay-image149765103
Philippines/ Manila Bay	Social Media	https://ichkaufnix.com/2016/07/16/der-plastikstrand-oder-wie-wir-uns-selbst-vergiften/
