# Supplementary material for: MARIDA: A benchmark for Marine Debris detection from Sentinel-2 remote sensing data
Source: PLoS One. 2022 Jan 7;17(1):e0262247. doi: 10.1371/journal.pone.0262247 (PMC8740969; doi:10.1371/journal.pone.0262247)
Supplement: S2 Table — (PDF) [file pone.0262247.s002.pdf]

S2 Table. The revisit time (days) of Sentinel-2 and Planet satellite sensors in study sites.

Country	Sentinel-2	Planet
Honduras/ Guatemala/ Haiti	2-5	1-2
Scotland	2-3	1-3
South Africa	2-5	1-3
Santo Domingo	5	1-3
Philippines/ Indonesia/ S.Korea	5	1-2
China/ Vietnam	5	1-5
