# Supplementary material for: MARIDA: A benchmark for Marine Debris detection from Sentinel-2 remote sensing data
Source: PLoS One. 2022 Jan 7;17(1):e0262247. doi: 10.1371/journal.pone.0262247 (PMC8740969; doi:10.1371/journal.pone.0262247)
Supplement: S3 Table — Corresponding Planet data (photo-interpretation process) are presented. (PDF) [file pone.0262247.s003.pdf]

S3 Table. The acquisition dates (day/month/year) of Sentinel-2 satellite data used for MARIDA construction. Corresponding Planet data (photo-interpretation process) are presented.

Country	S2 Tile	S2	Planet
**Guatemala**	16PCC	4/9/2016	29/8/2016
		12/1/2017	12/1/2017
		21/2/2017	-
		30/8/2017	24/8/2017, 30/8/2017
		21/2/2018	-
		26/2/2018	-
		11/6/2018	11/6/2018, 19/6/2018
		30/8/2018	30/8/2018
		14/9/2018	11/9/2018
		19/9/2018	19/9/2018
		13/12/2018	13/12/2018
		27/1/2019	-
		4/9/2019	4/9/2019
		24/8/2020	24/8/2020, 26/8/2020
		18/9/2020	18/9/2020
		23/9/2020	23/9/2020
		28/9/2020	28/9/2020
		15/11/2020	15/11/2020
		12/12/2020	11/12/2020
**Honduras**	16PDC	3/11/2016	-
		19/9/2018	19/9/2018
		24/10/2018	24/10/2018, 25/10/2018
		3/11/2018	3/11/2018, 6/11/2018
		18/9/2020	18/9/2020, 22/9/2020
		28/9/2020	28/9/2020
	16PEC	29/11/2015	24/11/2015
		12/1/2017	-
		9/10/2017	9/10/2017, 8/10/2017
		16/2/2018	15/2/2018, 16/2/2018
		8/3/2018	8/3/2018
		12/1/2019	-
	16QED	8/3/2018	8/3/2018
		27/1/2019	27/1/2019
**Haiti**	18QWF	22/3/2020	22/3/2020, 24/3/2020
	18QYF	14/3/2020	14/3/2020
		19/3/2020	19/3/2020
		24/3/2020	24/3/2020
		15/9/2020	15/9/2020
		15/10/2020	15/10/2020
		20/10/2020	19/10/2020
		29/11/2020	29/11/2020
		4/12/2020	4/12/2020
		14/12/2020	14/12/2020
		22/12/2020	22/12/2020
		29/12/2020	29/12/2020
		3/1/2021	3/1/2021
		23/1/2021	21/1/2021, 23/1/2021
	18QYG	7/3/2020	5/3/2020
**S. Domingo**	19QDA	11/1/2019	-
**Scotland**	30VWH	20/4/2018	20/4/2018
**S. Africa**	36JUN	24/4/2019	25/4/2019
**Indonesia**	48MXU	6/12/2018	6/12/2018
		25/5/2019	25/5/2019
	48MYU	6/12/2017	6/12/2017
		1/12/2019	3/12/2019
	50LLR	4/3/2018	4/3/2018
**Philippines**	51PTS	17/7/2016	17/7/2016
		18/5/2019	18/5/2019
**China**	51RVQ	29/8/2017	-
**S. Korea**	52SDD	7/10/2018	7/10/2018
**Vietnam**	48PZC	18/1/2018	18/1/2018
		14/11/2018	14/11/2018
		24/11/2019	23/11/2019
