# Supplementary material for: MARIDA: A benchmark for Marine Debris detection from Sentinel-2 remote sensing data
Source: PLoS One. 2022 Jan 7;17(1):e0262247. doi: 10.1371/journal.pone.0262247 (PMC8740969; doi:10.1371/journal.pone.0262247)
Supplement: S4 Table — (PDF) [file pone.0262247.s004.pdf]

S4 Table. The distribution of the confidence scores in pixel level for the classes of Marine Debris, Natural Organic Material and Sparse Sargassum.

**Confidence Level**	**Marine Debris**	**Natural Organic Material**	**Sparse *Sargassum***
High	1625 (47.81%)	556 (64.35%)	2052 (87.06%)
Moderate	1235 (36.33%)	201 (23.26%)	290 (13.3%)
Low	539 (15.86%)	107 (12.38%)	15 (0.64%)
**Total**	**3399**	**864**	**2357**
