# Supplementary material for: MARIDA: A benchmark for Marine Debris detection from Sentinel-2 remote sensing data
Source: PLoS One. 2022 Jan 7;17(1):e0262247. doi: 10.1371/journal.pone.0262247 (PMC8740969; doi:10.1371/journal.pone.0262247)
Supplement: S5 Table — All acronyms are stated in Table 2. (PDF) [file pone.0262247.s005.pdf]

S5 Table. Class distribution (%) for each split in MARIDA. All acronyms are stated in Table 2.

Class	Train	Validation	Test	Total
MD	27.38	25	28.13	27.01
DenS	3.46	3.66	3.62	3.55
SpS	8.07	7.93	6.69	7.68
NatM	5.04	6.1	4.46	5.14
Ship	12.82	12.8	14.21	13.18
Cloud	13.4	13.11	12.53	13.11
MWater	62.25	64.63	62.95	63
SLWater	3.46	4.27	3.62	3.69
Foam	4.32	4.27	4.18	4.27
TWater	16.57	15.24	15.32	15.93
SWater	5.48	3.35	4.18	4.63
Waves	3.31	5.49	3.62	3.91
CloudS	5.62	5.79	3.62	5.14
Wakes	7.2	8.23	8.08	7.68
MixWater	9.94	10.67	10.03	10.14
