# Supplementary material for: MARIDA: A benchmark for Marine Debris detection from Sentinel-2 remote sensing data
Source: PLoS One. 2022 Jan 7;17(1):e0262247. doi: 10.1371/journal.pone.0262247 (PMC8740969; doi:10.1371/journal.pone.0262247)
Supplement: S6 Table — All acronyms are stated in Table 2. (PDF) [file pone.0262247.s006.pdf]

S6 Table. Evaluation scores obtained by ResNet for each class on multi-label classification. All acronyms are stated in Table 2.

Class	F_1
MD	0.46
DenS	0.00
SpS	0.00
NatM	0.00
Ship	0.04
Cloud	0.46
MWater	0.87
SLWater	0.77
Foam	0.11
TWater	0.67
SWater	0.13
mF1 (Average)	**0.32**
Coverage Error	**6.93**
