# Supplementary material for: MARIDA: A benchmark for Marine Debris detection from Sentinel-2 remote sensing data
Source: PLoS One. 2022 Jan 7;17(1):e0262247. doi: 10.1371/journal.pone.0262247 (PMC8740969; doi:10.1371/journal.pone.0262247)
Supplement: S1 Appendix — (PDF) [file pone.0262247.s007.pdf]

S1 Appendix: Cloud and floating material annotation.

Regarding our annotation strategy, clouds/thin clouds were determined by their intensity, texture and shape, as well as by their adjacent shadows. Regarding thin clouds, following the same manner as for the other features/ classes, spectral signatures were examined, and confidence levels were assigned. After the inter-agreement protocol, the union of the annotations was estimated, while in cases with different confidence level labelling, the lowest confidence level was kept (we note that #1 is for high confidence level, #2 for moderate and #3 for low confidence level).

In cases that floating materials were below thin clouds or semi-transparent features such as aerosols or haze, the experts proceeded with the annotation of the considered floating feature only when its spectral profile/ shape/ pattern was not significantly distorted (assigning also confidence levels in all cases).
