# Supplementary material for: MARIDA: A benchmark for Marine Debris detection from Sentinel-2 remote sensing data
Source: PLoS One. 2022 Jan 7;17(1):e0262247. doi: 10.1371/journal.pone.0262247 (PMC8740969; doi:10.1371/journal.pone.0262247)
Supplement: S2 Appendix — (PDF) [file pone.0262247.s008.pdf]

S2 Appendix: The evaluation metrics

To evaluate the performance of RF model and U-Net architecture, we relied on the Intersection-over-Union (IoU):

$$IoU = \frac{TP}{TP + FP + FN}, \tag{1}$$

where TP is the number of true positives, FP the number of false positives and FN the number of false negatives. Intuitively, IoU is equal to the ratio of intersection between predicted and ground truth area to the union between predicted and ground truth area. Considering that we deal with a multi-class task, the main evaluation metric for the overall assessment is the mIoU which is the average IoU over all classes c:

$$mIoU = \frac{1}{n}\sum_{c}^{n} IoU_c \tag{2}$$

The second metric is the average for each class F_1 score (Macro-F_1/ mF_1), which is the harmonic mean between precision $P = TP/(TP + FP)$ and recall $R = TP/(TP + FN)$:

$$mF_1 = \frac{1}{n}\sum_{c}^{n} F_{1_c} = \frac{1}{n}\sum_{c}^{n} \frac{2P_c R_c}{P_c + R_c} \tag{3}$$

The third metric is the Pixel Accuracy (PA) for the per-class assessment and the corresponding mean PA (mPA) for the overall assessment. For each class, PA is the ratio of the correctly predicted pixels to the total number of pixels. For the multi-class pixel-level classification, this metric is equivalent to recall R.

To assess the performance of the multi-label classification task, we relied on the ranking-based metric Coverage error (Cov) [1], which is suitable for *weakly supervised* multi-label classification with unassigned labels. Cov estimates the average number of labels required to be included in final predictions of multi-label classifier such that all ground truth labels are predicted. Specifically, it is defined:

$$COV = \frac{1}{N_{samples}} \sum_{i=0}^{N_{samples}-1} \max_{j:y_{ij}=1} rank_{ij}, \tag{4}$$

where $y \in \{0, 1\}^{N_{samples} \times N_{labels}}$ are the ground truth labels, $\hat{s} \in \mathbb{R}^{N_{samples} \times N_{labels}}$ are the output scores, and $rank_{ij} = \left|\{k: \hat{s}_{ik} \geq \hat{s}_{ij}\}\right|$ is the number of scores greater than $\hat{s}_{ij}$ for the i sample and j label.

We also relied on the classification based Macro-F_1 score formulated in Eq. (3).

References

1. Tsoumakas G, Katakis I, Vlahavas I. Mining Multi-label Data. In: Maimon O, Rokach L, editors. Data Mining and Knowledge Discovery Handbook. Boston, MA: Springer US; 2010. pp. 667–685. doi:10.1007/978-0-387-09823-4_34
