# Supplementary material for: MARIDA: A benchmark for Marine Debris detection from Sentinel-2 remote sensing data
Source: PLoS One. 2022 Jan 7;17(1):e0262247. doi: 10.1371/journal.pone.0262247 (PMC8740969; doi:10.1371/journal.pone.0262247)
Supplement: S3 Appendix — (PDF) [file pone.0262247.s009.pdf]

S3 Appendix: Class weighting scheme

To address the unbalanced data limitation, the proposed weighting scheme by Paszke et al. [1] was used on the Cross-Entropy loss for each class:

$$w_{class} = \frac{1}{\ln(c + p_{class})}, \tag{5}$$

where $p_{class} = N_{pixels\,of\,class} / N_{total\,pixels}$ in the training set and c is an additional hyper-parameter, which was set to 1.03.

References:

1. Paszke A, Chaurasia A, Kim S, Culurciello E. ENet: A Deep Neural Network Architecture for Real-Time Semantic Segmentation. arXiv:160602147 [cs]. 2016. Available: http://arxiv.org/abs/1606.02147
