# Supplementary material for: MARIDA: A benchmark for Marine Debris detection from Sentinel-2 remote sensing data
Source: PLoS One. 2022 Jan 7;17(1):e0262247. doi: 10.1371/journal.pone.0262247 (PMC8740969; doi:10.1371/journal.pone.0262247)
Supplement: S4 Appendix — (PDF) [file pone.0262247.s010.pdf]

S4 Appendix: ResNet baseline

Regarding ResNet, we used the version with 50 layers (ResNet-50). Similar to the U-Net, we modified the first and final layers to adapt to MARIDA. To minimize Binary Cross-Entropy loss, we employed the Adam algorithm with an initial learning rate of 2×10^{-4} with a weighted regularization term of 10^{-6} (L2 penalty). We trained for 18 epochs, and at the 5th, 10th and 15th epochs, the learning rate was reduced by a 0.2 factor. The selected batch size was 32 samples. We utilized the same data augmentation techniques as in U-Net training and trained the ResNet from scratch. To reduce the bias for each class between the number of positive and negative samples, it is a common practice to use as weights the fraction $w = N_{negative}/N_{positive}$. However, we found it useful to normalize these weights to approx. 0-2 interval using Eq. (5) in S3 Appendix. In particular, we modified p_{class} as $p_{class} = 1/w$ and set $c = 1.6$.
