# Supplementary material for: MARIDA: A benchmark for Marine Debris detection from Sentinel-2 remote sensing data
Source: PLoS One. 2022 Jan 7;17(1):e0262247. doi: 10.1371/journal.pone.0262247 (PMC8740969; doi:10.1371/journal.pone.0262247)
Supplement: S5 Appendix — (PDF) [file pone.0262247.s011.pdf]

S5 Appendix: Features correlation and importance

In order to inspect our developed model with the best performance ($RF_{SS+SI+GLCM}$), and subsequently understand the importance of each input feature, we applied the permutation feature importance. Specifically, for each feature, we performed random shuffling between different samples on test set to obtain the amount of decrease in the model's accuracy. For each feature, we performed this procedure 20 repeated times to estimate the mean decrease. However, due to the multicollinearity of the input features, permuting one feature does not affect model's accuracy as long as similar information is still included. Thus, we calculated the Spearman Correlation among input features on train set, in order to form highly correlated groups and keep only a single feature from each group (S1 Fig).

Based on S1 Fig, we selected a cut-off threshold to form the groups with the most correlated features. Therefore, we selected 440 nm, 560 nm, 1600 nm, NDVI, FAI, FDI, SI, NDWI, NDMI, CON, HOMO and COR, each of which represents a different group. Followingly, we trained the $RF_{SS+SI+GLCM}$ from scratch only on these features and obtained almost identical results (mIoU = 0.67, mPA = 0.79, mF_1 = 0.78), compared to initial approach. This fact reveals that the selected subset of features preserves the same amount of input information. Finally, we used the permutation feature importance for this model and obtained the results demonstrated in Fig 6. We observe that the largest accuracy decrease occurs by permuting CON, NDWI, NDVI and FDI.
