# Supplementary figures and images for: MARIDA: A benchmark for Marine Debris detection from Sentinel-2 remote sensing data
Source: PLoS One. 2022 Jan 7;17(1):e0262247. doi: 10.1371/journal.pone.0262247 (PMC8740969; doi:10.1371/journal.pone.0262247)

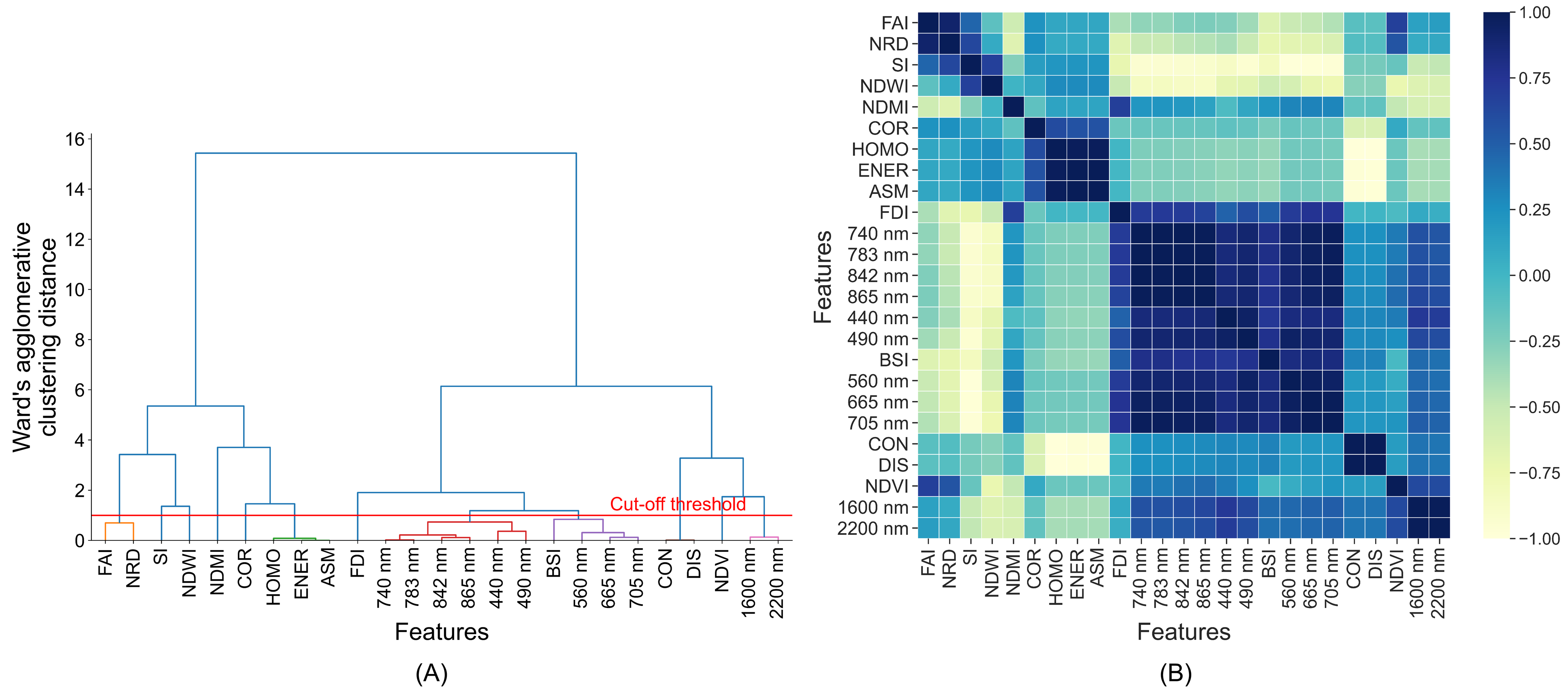

Supplement: S1 Fig — (A) Agglomerative hierarchical clustering on Spearman Correlation. (B) Heatmap of features correlation. (TIF) [file pone.0262247.s012.tif]
